# The Therapeutic Implications of the Gut Microbiome and Probiotics in Patients with NAFLD

**DOI:** 10.3390/diseases7010027

**Published:** 2019-02-25

**Authors:** Brandon J. Perumpail, Andrew A. Li, Nimy John, Sandy Sallam, Neha D. Shah, Waiyee Kwong, George Cholankeril, Donghee Kim, Aijaz Ahmed

**Affiliations:** 1 College of Medicine, Drexel University, Philadelphia, PA 19129, USA; brandonperumpail@gmail.com; 2 Department of Medicine, Stanford University School of Medicine, Stanford, CA 94305, USA; andrewli@stanford.edu; 3 Division of Gastroenterology and Hepatology, Stanford University School of Medicine, Stanford, CA 94305, USA; nionnj@gmail.com (N.J.); SSallam@stanfordhealthcare.org (S.S.); NeShah@stanfordhealthcare.org (N.D.S.); WKwong@stanfordhealthcare.org (W.K.); georgetc@stanford.edu (G.C.); dhkimmd@stanford.edu (D.K.)

**Keywords:** NAFLD, NASH, nonalcoholic fatty liver disease, gut microbiome, probiotics, synbiotics

## Abstract

Recent breakthrough in our understanding pertaining to the pathogenesis of nonalcoholic fatty liver disease (NAFLD) has pointed to dysregulation or derangement of the gut microbiome, also known as dysbiosis. This has led to growing interest in probiotic supplementation as a potential treatment method for NAFLD due to its ability to retard and/or reverse dysbiosis and restore normal gut flora. A thorough review of medical literature was completed from inception through July 10, 2018 on the PubMed database by searching for key terms such as NAFLD, probiotics, dysbiosis, synbiotics, and nonalcoholic steatohepatitis (NASH). All studies reviewed indicate that probiotics had a beneficial effect in patients with NAFLD and its subset NASH. Results varied between studies, but there was evidence demonstrating improvement in liver enzymes, hepatic inflammation, hepatic steatosis, and hepatic fibrosis. No major adverse effects were noted. Currently, there are no guidelines addressing the use of probiotics in the setting of NAFLD. In conclusion, probiotics appear to be a promising option in the treatment of NAFLD. Future research is necessary to assess the efficacy of probiotics in patients with NAFLD.

## 1. Introduction

Nonalcoholic fatty liver disease (NAFLD) and its progressive subset, nonalcoholic steatohepatitis (NASH), affect a significant proportion of the population. However, no medications are currently approved for the treatment of NAFLD; the primary therapeutic intervention is lifestyle modification through diet and exercise [1,2,3]. The focus of this article is to review the role of the gut microbiome and probiotics in patients with NAFLD and its progressive subset nonalcoholic steatohepatitis (NASH).

Recent research has linked changes in the gut microbiome with the pathogenesis of NAFLD [4]. The ability of probiotics to re-establish a natural and healthy gut microbiome makes them a promising therapeutic option for patients with NAFLD; however, the American Association for the Study of Liver Disease (AASLD) and the European Association for the Study of the Liver (EASL) currently have no guidelines regarding the role of probiotics in patients with NAFLD and/or NASH [2]. 

## 2. The Gut Microbiome

The assortment of all the microorganisms that dwell on and inside our body is collectively known as the human microbiome [31]. This collection is so large that it outnumbers human cells by a factor of 10 [31]. Most of this is the gut microbiome which consists of the community of microorganisms that inhabit the digestive tract, hosting over 1000 species and their millions of genes [31]. It is often called the “forgotten organ”, due to its crucial role in physiology in healthy humans, but lack of in-depth exploration and research until recent development [31]. Furthermore, the gut microbiome community, comprised mostly of a balance between microorganisms of the phyla *Firmicutes*, *Bacteroidetes*, and *Actinobacteria* (in healthy adults), is imperative for the proper functioning and regulation of the immune system, detoxification, and digestion [31]. However, many factors can affect and change the composition of our gut microbiome such as age, illness, diet, hormonal changes, travel, and therapy [31]. The gut microbiome has been linked to many diseases, including obesity, cardiovascular disease, inflammatory bowel disease, malnutrition, osteoporosis, colorectal cancer, *Clostridium difficile* infection, and type 2 diabetes [32].

### 2.1. Gut Dysbiosis in NAFLD Patients

When the gut microbiome changes from that of a healthy individual and is impaired or imbalanced, this change is called dysbiosis. This change can consist of overgrowth or lack of growth of certain microorganisms such that the species that are normally the majority or predominant are outcompeted with and are diminished. In NAFLD and NASH patients, it has recently been noted that they have an altered gut microbiome [5,11,12,14,16,17,20,22,24,25,27]. In a study by Zhu et al., the microbiomes of healthy, obese, and NASH patients were compared with 16S ribosomal RNA sequencing and differences at the phylum, family, and genus level were noted between the healthy and the other two groups [5]. The obese and NASH patients were observed to have different ratios of *Firmicutes*, *Bacteroidetes*, and *Actinobacteria* compared to the healthy patients and had increased disease-causing bacterial strains to replace them such as *Clostridium* [5]. The NASH patients also had increased alcohol-producing bacteria which increased serum alcohol levels and oxidative stress [5]. Liu et al. noted that NAFLD-causing high-fat diets can also alter the gut microbiome to enact NAFLD pathogenesis in a study that fed different diets to mice and resulted in the high-fat diet-fed mice to have microbiomes similar to those in mice with NAFLD [20]. Similar changes in gut microbiome for pediatric patients were detected by Del Chierico et al. when he compared NAFLD, NASH, and obese pediatric patients with healthy controls [24]. The NAFLD patients had increased levels of *Anaerococcus*, *Bradyrhizobium*, *Dorea*, *Peptoniphilus*, *Propionibacterium acnes*, and *Ruminococcus* and reduced proportions of *Oscillospira* and *Rikenellaceae* compared to the healthy controls, and did not differ greatly from NASH and obese pediatric patients [24]. Moreover, Boursier et al. concluded that the gut microbiome dysbiosis can be used as a predictor of NAFLD severity and progression [27]. In their 57-NAFLD-patient study, it was determined through multivariate analysis that the *Bacteroides* is independently associated with NASH, and *Ruminococcus* with fibrosis [27]. Although there has not been definitive research indicating a direct causative relation, the next section indicates the possible pathways through which these various bacterial strains can lead to NAFLD pathogenesis. 

### 2.2. Gut Dysbiosis in NAFLD Pathogenesis

Current research has indicated that there is a clear association between the dysbiosis of the gut and occurrence of NAFLD; however, future clarity is necessary as to whether this relation is causal and the mechanism by which dysbiosis plays a role in NAFLD pathogenesis. The major proposed pathways of disease from dysbiosis are through the gut–liver axis resulting in increased inflammation, steatosis, and fibrosis [25]. Inflammation occurs mostly through the activated toll-like receptors (TLRs) of various hepatic cells [18]. These TLRs are normally inactive in healthy liver cells and are pattern recognition receptors that bind pathogen-associated molecular patterns (PAMPs) and damage-associated molecular patterns (DAMPs) [21]. These molecules enter the liver through the portal circulation and can activate the TLRs causing an inflammatory response in the liver [18]. Lipopolysaccharide (LPS) is an endotoxin bacterial component that is one of the most prominent TLR activators (TLR4) and can initiate the inflammatory cascade from Kupffer cells and inflammasome activation [18]. The increased LPS levels in the hepatic portal circulation due to the dysbiotic state leads to increased lipopolysaccharide-binding protein (LBP) and endotoxin levels in patients and NAFLD progression through increased inflammation and fibrosis [15,16,18]. Higher levels of LBP, in particular, were shown to be associated with NASH as compared to NAFLD, and both LBP and endotoxin levels are correlated with increasing fibrosis as measured by transient elastography [15]. In another study, repeated LPS injections in ob/ob mice led to steatohepatitis [33]. Other bacterial components such as bacterial DNA, peptidoglycan, and flagellin can activate TLR2, TLR5, and TLR9, which have also been extensively studied as NAFLD inflammatory agents [9,10,18]. Furthermore, TLR activation is intensified by increased intestinal permeability due to the state of dysbiosis [14,22]. The intestinal barrier that allows for immunological tolerance of hepatocytes (especially Kupffer cells) from PAMPs and DAMPs is disrupted [14]. This occurs primarily through the effects of ethanol from introduced alcohol-producing bacteria (*Escherichia*) as well as toxins from *Clostridia* bacteria that bind to tight junction proteins and prevent their function [5,7,30]. Also, the increased endogenous alcohol production from the bacteria increases hepatic alcohol metabolism, ROS production, and oxidative stress, leading to hepatic inflammation [5]. 

Endogenous ethanol production has been linked with increased hepatic activity of cytochrome P450-2E1 (CYP2E1) [34]. CYP2E1 has high catalytic activity for alcohol [35], in the process generating significant amounts of free radicals and reactive oxygen species that subsequently cause cellular damage, mitochondrial dysfunction, and hepatic inflammation [34,36,37,38,39]. In NAFLD and NASH specifically, CYP2E1 has also been shown to be induced by high-fat diets in rats [40], elevating oxidative and nitrosative stress that contribute to NASH, with Cyp2e1-null mice being somewhat protected [41]. In humans, CYP2E1 expression and activity are increased in patients with NAFLD and are even higher in patients with NASH [42]. Beyond the liver, there is data to suggest that intestinal CYP2E1 plays a role in mediating alcohol-induced gut leakiness in a chronic alcohol use model [43]. However, the interplay between CYP2E1, the microbiome, and NAFLD is still poorly understood, with further studies required to elucidate the effects of the gut microbiota on intestinal and hepatic CYP2E1 and eventually the pathogenesis of NAFLD and NASH.

Furthermore, gut dysbiosis can increase steatosis through metabolic modulation [14]. The bacterial fermentation of indigestible carbohydrates and proteins allows for the production of short-chain fatty acids (SCFAs) that act as energy sources and signaling molecules in the human body [13]. These SCFAs, mainly butyrate, propionate, and acetate, account for approximately 30% of the energy extracted from the diet [8]. Of the three, acetate is produced by *Bacteroidetes*, is absorbed the most, and is obesogenic, causing lipogenesis and cholesterol synthesis in adipose tissue and the liver [19]. Butyrate, on the other hand, is considered to have anti-obesogenic effects by increasing insulin sensitivity, improving gut permeability, and increasing leptin [14]. However, in dysbiosis of the gut, there are changes in the ratio of *Bacteroidetes* to *Firmicutes*, and increased acetate production and overall SCFA production that promote steatosis [14]. Steatosis is also increased during the state of dysbiosis due to its inhibitory effect on fasting-induced adipose factor (FIAF) and adenosine monophosphate-activated protein kinase (AMPK) [28,29] FIAF inhibition results in increased lipoprotein lipase activity and fatty acid uptake by adipose tissue and the liver, while AMPK inhibition leads to decreased peripheral fatty acid oxidation and adipose accumulation [28,29]. The metabolism of bile acids that are crucial for fat/fat-soluble vitamin digestion and absorption as well as signaling through the farnesoid X receptor (FXR) and the G-protein coupled bile salt receptor 1 (GBAR1 or TGR5) is affected during the dysbiotic state [6]. Its signaling effects alter hepatic lipogenesis, gluconeogenesis, very-low-density lipoprotein (VLDL) export, glycogen synthesis, bile acid production, and insulin sensitivity which can lead to hepatic inflammation, oxidative stress, and even fibrosis [23]. Finally, choline metabolism is also disrupted in dysbiosis [26]. Choline is crucial for VLDL synthesis and hepatic lipid export; however, in dysbiosis the microorganisms in the gut metabolize choline to trimethylamine (TMA) which is oxidized in the liver to become trimethylamine-*N*-oxide (TMAO) [26]. This leads to hepatic inflammation, fibrosis, and steatosis due to alterations in hepatic glucose and lipid metabolism [26]. Although dysbiotic changes are noted in NAFLD patients and they adversely affect the liver, further studies are necessary to determine the relationship between gut dysbiosis and NAFLD. It is not clearly understood whether gut dysbiosis causes NAFLD particularly or if the lifestyle and diet associated with NAFLD in patients also leads to gut dysbiosis. 

### 2.3. The Proposed Therapeutic Role of Probiotics and Animal Trials 

These dysbiotic changes observed in NAFLD patients indicate the possible role of the gut microbiome in NAFLD pathogenesis, making it an optimal target for drug intervention—probiotics. These are live microorganisms that when consumed or applied in sufficient quantity confer health benefits to the host [44]. Thus, by definition they must improve the health of the host to be considered a probiotic [44]. Furthermore, probiotics must be able to survive the transit to the gut, as well as be able to grow and profligate in the gut to confer its benefits to the host [44]. To counteract dysbiosis seen in NAFLD patients, probiotics can normalize gut microbiota to the healthy state and reverse adverse gut microbiota changes to allow for proper function of the system physiology [44]. 

**Figure 1:**
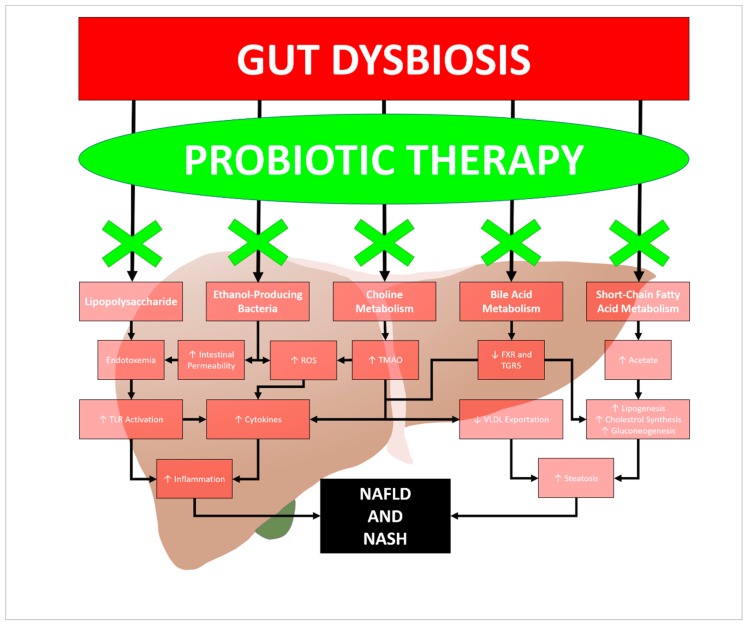
The proposed role of probiotics in NAFLD therapy [5,6,7,8,9,10,11,12,13,14,15,16,17,18,19,20,21,22,23,24,25,26,27,28,29,30]. ROS = reactive oxygen species; TMAO = trimethylamine N-oxide; FXR = farnesoid X receptor; TGR5 = G-protein coupled bile salt receptor 1; TLR = toll-like receptor; VLDL = very-low-density lipoprotein; NAFLD = non-alcoholic fatty liver disease; NASH = non-alcoholic steatohepatitis.

Multiple experimental trials have shown therapeutic effects of probiotics in mice as well. Xin et al. conducted a trial with mice fed a high-fat diet, where the administration of the probiotic *Lactobacillus johnsonii BS15* was able to prevent the onset hepatic steatosis and apoptosis by improving hepatic inflammation and oxidative stress [45]. Another experimental trial was conducted by Liang et al. who gave compound probiotics with a high-fat diet to mice with NAFLD, and noted that the mice had improved gut dysbiosis, as well as hepatic lipid deposition [46]. Improved fibrosis and decreased endotoxemia were observed in an experimental trial with NAFLD-induced mice given probiotics by Cortez-Pinto et al. [47]. The most studied probiotic therapy used with NAFLD in which the pathway of action has been clearly validated is the VSL#3 bacterial blend [48]. It is a multispecies therapy that uses eight strains of bacteria (*Lactobacillus plantarum*, *Lactobacillus delbrueckii*, *Lactobacillus casei*, *Lactobacillus acidophilus*, *Bifidobacterium breve*, *Bifidobacterium longum*, *Bifidobacterium infantis*, and *Streptococcus thermophilus*) [48]. In mice trials, VSL#3 has been indicated to have anti-inflammatory effects through the modulation of nuclear factor kappa B (NF-κB) and tumor necrosis factor (TNF), as well as antifibrotic effects through modification of transforming growth factor beta (TGF-β) and collagen expression [49,50]. These positive findings in experimental studies suggest that probiotic therapy may have a therapeutic effect on NAFLD patients, though experiments with germ-free animal models would help better elucidate the mechanisms by which they confer their beneficial advantages. Figure 1 discusses the proposed role of probiotics as a method to reverse pathogenic effects associated with the dysbiotic state observed in NAFLD patients.

## 3. Clinical Use of Probiotics in Patients with NAFLD and NASH 

Not many clinical studies have been conducted to explore the role of probiotics as a possible treatment therapy for NAFLD and NASH patients, due to its novelty as a treatment method. Various means were used to measure the results—biochemical parameters (liver enzyme levels) and hepatic histology (steatosis, lobular inflammation, balloon degeneration, and fibrosis). This distinction is important because although liver enzymes are commonly used to assess the improvement in NASH, they are poor predictors of NASH and its prognosis. These clinical studies explored the efficacy of probiotics, as well as alternative probiotic approaches. Various other therapies include prebiotics and synbiotics. Prebiotics are used to nourish healthy bacteria and allow for better efficacy of probiotic therapy. At their introduction by Gibson and Roberfroid, prebiotics were described as a “non-digestible food ingredient that beneficially affects the host by selectively stimulating the growth and/or activity of one or a limited number of bacteria in the colon”—essentially growth agents for probiotics [51]. Synbiotics are the combined use of prebiotics and probiotics [44]. The studies clearly show that probiotics have a positive effect on the various measures of NAFLD in patients; however, the mechanism by which this occurs needs further elucidation. 

### 3.1. Biochemical Studies 

At least six clinical studies that assessed serum levels of aspartate aminotransferase (AST) and alanine aminotransferase (ALT) to evaluate liver function demonstrated significant improvement with probiotic and symbiotic treatment [52,53,54,55,56,57]. An early study by Aller et al. shed light on the usage of a 500-million *Lactobacillus bulgaricus* and *Streptococcus thermophilus* tablet in NAFLD patients per day versus a placebo tablet daily, and noted significant improvement in both liver function enzymes in the probiotics group and no change in the control [52]. Another double-blinded trial by Wong et al. randomly divided 20 NASH patients into control and probiotics groups, the latter of which received the Lepicol probiotic formula containing *Lactobacillus plantarum*, *Lactobacillus bulgaricus*, *Lactobacillus acidophilus*, *Lactobacillus rhamnosus* and *Bifidobacterium bifidum* for six months, and noted improved AST in patients with probiotics, and proposed that it could possibly improve steatosis in future studies [54]. A 72-NAFLD-patient double-blind study by Nabavi et al. compared treatment with 300g/d of probiotic yogurt with *Lactobacillus acidophilus La5* and *Bifidobacterium lactis Bb12* versus conventional yogurt and noted improved hepatic enzymes, total cholesterol, and LDL cholesterol [55]. International studies have also observed similar results [56]. An Egyptian study by Abdel Monem with Zigazag University randomized NASH patients to probiotic *Acidophilus* capsule (2 billion *Lactobacillus acidophilus*) or placebo for 17 months and measured improved AST and ALT in treated patients [56]. Wang et al. compared the effectiveness of various probiotics with a control in 200 NAFLD patients and measured significantly improved liver function tests in all probiotics groups versus the control, but no significance between them [57]. The treatment groups were *Bifidobacterium Lactobacillus* and *Enterococcus powder*, *Bacillus subtilis* and *Enterococcus powder*, or a combination therapy of both probiotics [57]. Interestingly, the combination therapy was no better or worse than either individual therapy [57]. Meta-analysis of studies with biochemical measurements also concluded that probiotics were beneficial [58,59]. Both studies calculated improvement in liver enzymes, total cholesterol, and inflammatory factors [58,59]. 

### 3.2. Histological Studies

Several studies measured hepatic histology in their evaluation of the efficacy of probiotics including assessment of hepatic inflammation, steatosis, and fibrosis [60,61,62,63,64,65,66,67]. A pilot study by Elsamparast et al. studied the effects of synbiotics in addition to lifestyle medication versus lifestyle modification alone in NAFLD patients [62]. The results of this study revealed that symbiotic supplementation is superior to lifestyle modification alone at improving liver enzymes and fibrosis in patients [62]. Another trial by Manzhalii et al. randomized NASH patient receiving low-calorie/low-fat diet to receive a placebo or oral combination of *Lactobacilli*, *Bifidobacteria* and *Streptococcus thermophilus* for 12 weeks [64]. Patients receiving probiotics had reduced ALT, hepatic inflammation, and liver stiffness [64]. Stool analysis of the treated patients revealed a shift in the gut microbiome towards normal composition [64]. Mofdi et al. randomized NAFLD patients to receive symbiotic treatment or placebo for 28 weeks [65]. At the end of the study, the treated patients exhibited not only decreased inflammatory markers, but also significantly decreased hepatic fibrosis and steatosis [65]. A 24-week trial with 102 NAFLD patients by Bakhshimoghaddam et al. randomized patients into three groups—one control and two interventions (300 g synbiotic yogurt containing 108 colony-forming units *Bifidobacterium animalis* or conventional yogurt) [66]. Ultrasonography graded NAFLD scores were significantly decreased in the synbiotic group, as well as AST, ALT, and hepatic steatosis compared to the other groups [66]. A double-blinded single-center trial treated NAFLD patients with the multi-probiotic "Symbiter", a concentrated biomass of 14 different strains of probiotic bacteria genera *Bifidobacterium*, *Lactobacillus*, *Lactococcus*, *Propionibacterium*, and *Acetobacter*, and compared results against a control after eight weeks [67]. Patients treated with the multistrain probiotic exhibited decreased fatty liver indices (decreased steatosis), decreased liver enzymes, and decreased inflammatory markers [67]. Several studies have indicated that probiotics are also effective in pediatric and adolescent patients as well [61,63]. Alisi et al. studied the effects of VSL#3, a commonly used probiotic for irritable bowel syndrome that contains eight strains of *Bifidobacterium* and *Lactobacillis*, in obese NALFD pediatric patients, and noted decreased steatosis and improved liver enzymes after four months of treatment [61]. Faouri et al. used a probiotic capsule containing *Lactobacillus acidophilus*, *Bifidobacterium bifidum*, *Bifidobacterium lactis*, and *Lactobacillus rhamnosus* in pediatric and adolescent NAFLD patients and noted improved liver enzymes and normalized liver sonography after treatment [63]. An evidence-based case report pulled data from six critically appraised studies and concluded that probiotics reduce hepatic inflammation and liver enzymes [68]. Finally, meta-analysis of seven trials with probiotics and NAFLD concluded that therapy improved AST, ALT, and ultrasonic grade of liver steatosis [69]. Table 1 summarizes the clinical studies reviewed.

## 4. Safety and Tolerability of Probiotic Formulations

All studies reviewed reported no adverse effects or issues of safety with the clinical use of probiotics in patients with NAFLD [52,53,54,55,56,57,60,61,62,63,64,65,66,67]. In the evaluation of these studies, a potential fallback noted was the lack of proper time duration of the study to observe any long-term effects of probiotic use and whether the termination of probiotics reverted the gut microbiome to its previous state before treatment was initiated. A better understanding of the longer-term safety profile is of particular importance in NAFLD and NASH as patients often have multiple comorbid conditions that require multiple drugs, each potentially with the risk of drug-induced liver injury [70]. For example, there is growing evidence that the hepatotoxicity of drugs may be more frequent and severe when exposed to a fatty liver as in NAFLD. This has been seen with losartan, multiple antibiotics, statins, rosiglitazone, and acetaminophen [71,72]. It has been hypothesized that this higher risk may be in part related to increased activity of several cytochromes P450, including CYP2E1, in obese patients [73,74]. Clinical trials should, therefore, be cognizant of this and adjust for this, potentially using the updated Roussel Uclaf Causality Assessment Method (RUCAM) [75], as well as other environmental factors such as alcohol use and herbal use as potential confounding factors. 

The gut microbiome is a very robust system and it is difficult to determine the benefit modification without enduring research. Future studies should follow patients for a longer duration to determine the effects probiotic usage long term with ongoing usage and effects after the termination of therapeutic intervention. A concern about probiotic usage posed by Brandi et al. noted that the gut microbiome is very resilient and can return to its original organization and variation after temporary intervention [76]. This issue should be explored more thoroughly in future research testing the effects of probiotics as a therapeutic option in NAFLD patients. However, it is important to note that the Food and Drug Administration of the United States has not ruled on any probiotic use or confirmed any therapeutic use of them [77]. The European Food and Safety Association is the current regulator of probiotic marketing and has a list of approved strains for safety [44]. Despite this, research in probiotic therapy is still preliminary and verified adverse effects of probiotics have yet to be detailed. 

## 5. Conclusions

Recent findings have noted gut dysbiosis as a risk factor that may influence and promote the pathogenesis of NAFLD. The ability of probiotics to reverse gut dysbiosis has generated increasing interest in efforts to study probiotics as an alternative therapeutic option in patients with NAFLD and/or NASH. Although probiotics have been used for decades, only recently have they been explored as a treatment in the context of NAFLD and NASH. Preliminary data as presented above have been encouraging. Existing data suggest that by retarding and reversing dysbiosis, probiotics can improve hepatic inflammation, histology, and function as measured by biochemical markers, as well as in liver biopsy samples. Theoretically, probiotics can be used alone or in conjunction with other NAFLD-targeted therapies. However, potential interactions with other agents must be studied. Despite these promising emerging data, sufficient evidence is lacking for experts to recommend the clinical use of probiotics for gut microbiome modification in patients with NAFLD. In this growing area of NAFLD-related therapeutic research, further studies are necessary to more clearly elucidate the role of probiotics.

## Figures and Tables

**Table 1:** Major clinical studies using probiotic therapy for NAFLD/NASH.

Reference	*N*	Probiotic Strains Used	Duration	Compared to Control/Placebo	
				Biochemical Improvement	Histological Improvement
Aller [52]	14	*Lactobacillus bulgaricus* *Streptococcus thermophilus*	3 months	AST, ALT	N/A
Shavakhi [53]	32	*Lactobacillus acidophilus* *Lactobacillus casei* *Lactobacillus rhamnosus* *Lactobacillus bulgaricus* *Bifidobacterium breve* *Bifidobacterium longum* *Streptococcus thermophilus*	6 months	AST, ALT	N/A
Wong [54]	10	*Lactobacillus plantarum* *Lactobacillus bulgaricus* *Lactobacillus acidophilus* *Lactobacillus rhamnosus* *Bifidobacterium bifidum*	6 months	AST	N/A
Nabvi [55]	28	*Lactobacillus acidophilus* *Bifidobacterium lactis*	8 weeks	AST, ALT	N/A
Abdel Monem [56]	15	*Lactobacillus acidophilus*	17 months	AST, ALT	N/A
Wang [57]	150	*Bifidobacterium Lactobacillus* *Enterococcus* *Bacillus subtilis*	1 month	AST, ALT	N/A
Loguercio [60]	22	VSL #3	4 months	AST, ALT	N/A
Alisi [61]	22	VSL #3	4 months	NS	St
Eslamparast [62]	52	*Lactobacillus acidophilus* *Lactobacillus casei* *Lactobacillus rhamnosus* *Lactobacillus bulgaricus* *Bifidobacterium breve* *Bifidobacterium longum* *Streptococcus thermophilus*	7 months	AST, ALT	Fi
Famouri [63]	32	*Lactobacillus acidophilus* *Bifidobacterium lactis* *Bifidobacterium bifidum* *Lactobacillus rhamnosus*	12 weeks	AST, ALT	St
Manzhalii [64]	37	*Lactobacilli, Bifidobacteria* *Streptococcus thermophilus*	12 weeks	ALT	LI, Fi
Mofidi [65]	25	*Lactobacillus acidophilus* *Lactobacillus casei* *Lactobacillus rhamnosus* *Lactobacillus bulgaricus* *Bifidobacterium breve* *Bifidobacterium longum* *Streptococcus thermophilus*	28 weeks	AST, ALT	St, Fi
Bakhshimoghaddam [66]	34	*Bifidobacterium animalis*	24 weeks	AST, ALT	St
Kobyliak [67]	29	*Bifidobacterium* *Lactobacillus* *Lactococcus* *Propionibacterium* *Acetobacter*	8 weeks	AST	St

AST = aspartate aminotransferase; ALT = alanine aminotransferase; NS = not significant; N/A = not applicable; St = steatosis; LI = lobular inflammation; Fi = fibrosis.
